# Development of a Dispersive Liquid–Liquid Microextraction Procedure for Biodegradation Studies on Nonylphenol Propoxylates Under Aerobic Conditions

**DOI:** 10.1007/s11743-013-1479-8

**Published:** 2013-04-26

**Authors:** Agnieszka Zgoła-Grześkowiak

**Affiliations:** Institute of Chemistry and Technical Electrochemistry, Poznan University of Technology, Piotrowo 3, 60-965 Poznań, Poland

**Keywords:** Nonylphenol propoxylates, Biodegradation, Fluorescence detection, Tandem mass spectrometry, Dispersive liquid–liquid microextraction

## Abstract

Aerobic biodegradation behavior of nonylphenol propoxylates was investigated using dispersive liquid–liquid microextraction as a simple and fast technique for sample preparation. The developed method proved to be efficient for the isolation and concentration of nonylphenol propoxylates before their quantification with the use of high performance liquid chromatography. The primary biodegradation of nonylphenol propoxylates was approximately 80 % by 10 days after the beginning of the test. However, the biodegradation products which were identified with the use of mass spectrometric detection persisted for many days.

## Introduction

Nonylphenol propoxylates (NPPOs) are a relatively new class of man-made surface active agents. They can be used as a co-surfactant in coatings and printing ink compositions and in low-foaming wetting agents. The molecules of NPPOs consist of two parts, both of which are considered problematic to the environment. On the one hand free nonylphenol is known from its endocrine disrupting properties [1, 2] and on the other hand the propoxy chain is regarded as being poorly biodegradable [3–5]. Accordingly, biodegradation studies on these surfactants would be of great interest.

Polarity of NPPOs is low, which influences their properties including low solubility in water. Therefore, their biodegradation must be studied at relatively low concentrations which implies usage of sample concentration methods before instrumental analysis of NPPOs.

Several sample concentration methods are known and widely used in the analysis of surfactants. Among them, the most widely used techniques are both classical liquid–liquid extraction and solid-phase extraction [6–8]. These methods could be replaced by the use of microextraction techniques which were developed for easier and low cost sample concentration. Solid-phase microextraction, single-drop microextraction and hollow-fibre microextraction are widely used and known [9–11]. Nevertheless, new microextraction techniques still emerge. Among them, the newly developed dispersive liquid–liquid microextraction (DLLME) found wide interest [12, 13]. In DLLME, a mixture of solvents is used for extraction of analytes from a water matrix. This mixture contains typically tens of microliters of an extraction solvent in a few milliliters of a dispersion solvent. The water soluble dispersion solvent (usually acetone, acetonitrile or methanol) is used to finely disperse the water insoluble extraction solvent (typically chloroform, chlorobenzene or other chlorinated solvents) in five to ten milliliters of a water sample. The extraction solvent is then separated by centrifugation. Proper amounts of the selected solvents must be set experimentally, however, the final method is potentially very fast, simple and inexpensive.

The present paper is devoted to both the development of an analytical method for sample preparation and a biodegradation study of NPPOs. Isolation and concentration of analytes were achieved using a DLLME procedure. The isolated compounds were quantified by high-performance liquid chromatography using a phenyl-hexyl analytical column. The developed procedure was then employed in a biodegradation study of using a commercial mixture of NPPOs as the substrate. To the best of our knowledge, neither an analytical method for sample isolation and concentration nor a biodegradation study of this surfactant have been previously reported in the literature.

## Materials and Methods

### Reagents and Chemicals

A commercial mixture of nonylphenol polypropylene glycol ethers with an average propoxylation degree of 10 was obtained from Sasol (Johannesburg, South Africa) as NONFIX 11011. It is abbreviated as NPPOA10 for nonylphenol with an Average of 10 propylene oxide units in what follows. Nonylphenol was obtained from Sigma-Aldrich (St. Louis, MO, USA). MS-grade and HPLC-gradient grade methanol and acetonitrile were from Sigma-Aldrich. Water was prepared by reverse osmosis in a Demiwa system from Watek (Ledec nad Sazavou, The Czech Republic), followed by double distillation from a quartz apparatus. Only freshly distilled water was used.

All the reagents applied as the extraction solvents in the experiments were of analytical grade. Chloroform, carbon tetrachloride and trichloroethane were from Sigma-Aldrich. Trichloroethylene and tetrachloroethylene were from Merck (Darmstadt, Germany). Analytical grade acetonitrile, acetone, methanol and ethanol used as the dispersion solvents were obtained from J.T. Baker (Deventer, The Netherlands). Ammonium formate was purchased from Sigma-Aldrich. All reagents used for preparation of the test medium were purchased from POCh (Gliwice, Poland).

### Biodegradation Study (Modified OECD Screening Test)

Static screening test for ready biodegradability under aerobic conditions was performed based on the Organisation for Economic Co-operation and Development (OECD) method 301E (Modified OECD Screening Test) [14]. NPPOs with an average propoxylation degree of 10 were tested. A surfactant concentration of 0.5 mg L^−1^ was applied in the test. The medium used in the test consisted of yeast and mineral components (KH_2_PO_4_, K_2_HPO_4_, Na_2_HPO_4_·2H_2_O, NH_4_Cl, CaCl_2_, MgSO_4_·7H_2_O, FeCl_3_·6H_2_O, MnSO_4_·4H_2_O, H_3_BO_3_, ZnSO_4_·7H_2_O and (NH_4_)_6_Mo_7_O_24_) as proposed by the OECD [14]. River water from River Warta (Poznań, Poland) was used as an inoculum in the test. Typically it consists of mostly spherical bacteria (1.4 10^3^–5.5 10^3^ cells cm^−3^, 46–264 μg L^−1^) and lesser amounts of rod-shaped (0.5 10^3^–2.5 10^3^ cells cm^−3^, 19–63 μg L^−1^) and spiral bacteria (0.1 10^3^–2.4 10^3^ cells cm^−3^, 8–69 μg L^−1^) bacteria, in addition to filamentous bacteria (0.01 10^3^–0.06 10^3^ cells cm^−3^, 4–99 μg L^−1^) and traces of other bacteria [15]. The test was performed in 200 mL bottles. One bottle was prepared for each experimental point. The biodegradation test lasted for 72 days.

### Dispersive Liquid–Liquid Microextraction Procedure

A 6-mL water sample was placed in a 15-mL glass test tube with a conical bottom. Then, 2.5 mL of ethanol (dispersion solvent) containing 60 μL of tetrachloroethylene (extraction solvent) was injected rapidly into the sample solution using a 2.5-mL syringe. In this step, the extraction solvent was dispersed into the aqueous sample as very fine droplets and a cloudy solution was formed in the test tube. Then, the mixture was centrifuged for 10 min at 4,500 rpm. The sedimented phase was withdrawn with a 100-μL micro-syringe. The extract was evaporated with a gentle nitrogen purge at room temperature and reconstituted to 30 μL of methanol and injected into an HPLC column for analysis.

### HPLC-FD Analysis of Nonylphenol Propoxylates

A Summit chromatographic system from Dionex (Sunnyvale, CA, USA) consisting of a P580 A LPG gradient pump, an ASI-100 autosampler, an STH 585 oven and an RF 2000 fluorescence detector was used. The 10-μL samples were injected into a Halo phenyl-hexyl column (50 mm × 3 mm I.D.; 2.7 μm) from Advanced Materials Technology (Wilmington, DE, USA). The mobile phase employed in the analysis consisted of water and methanol at a flow rate of 1 mL min^−1^. The gradient elution program was as follows: 0 min. 75 % methanol; 20 min. 90 % methanol; 25 min. 90 % methanol at 35 °C. A pre-run time of 4 min was done before the next injection. Signal responses were measured by fluorescence detection at wavelengths set at 225 nm for excitation and 300 nm for emission.

**Fig. 1:**
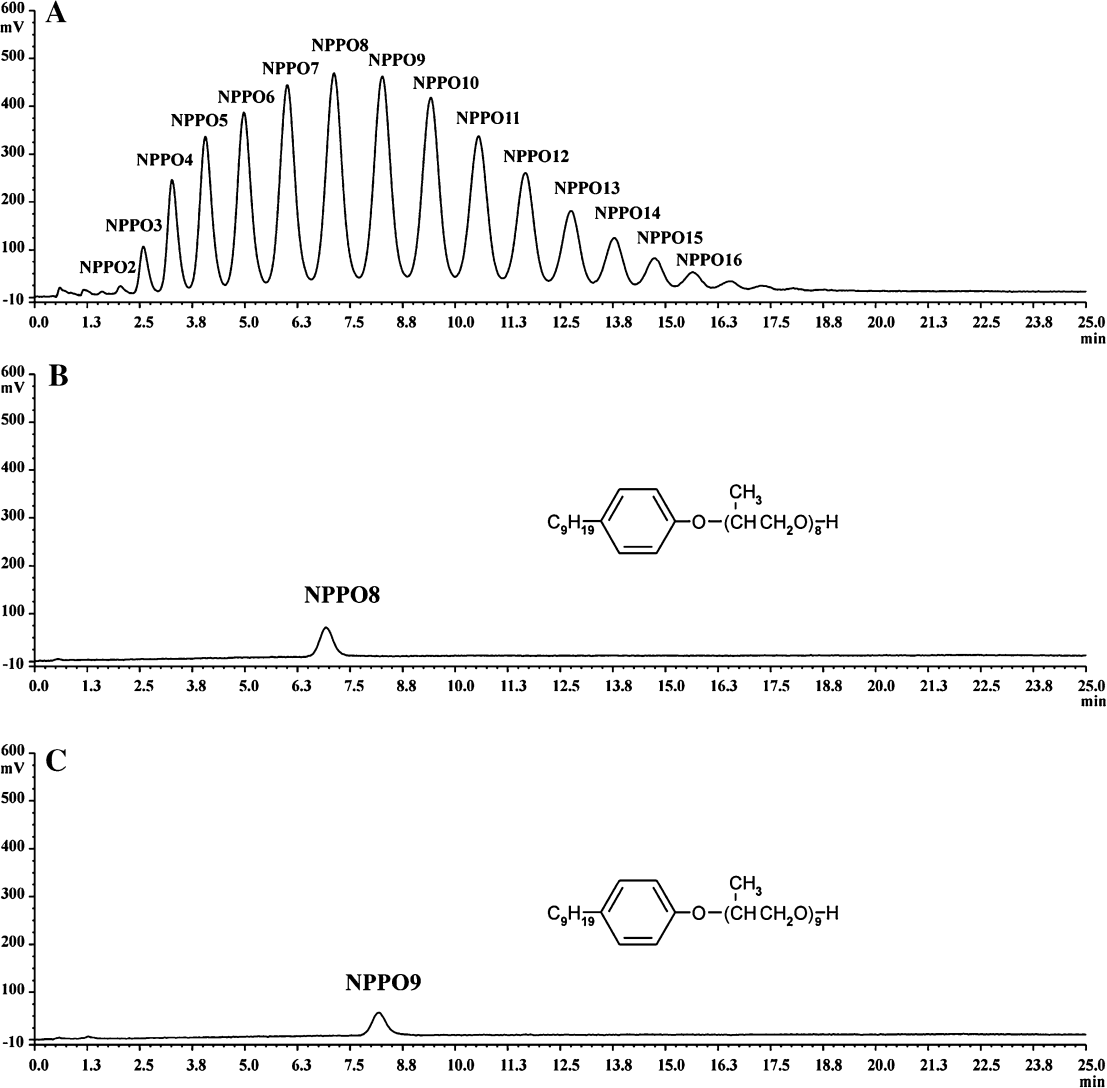
Chromatograms of nonylphenol propoxylates. **a** Separation of particular homologues belonging to NPPOA10 used in the biodegradation test, **b** isolated nonylphenol propoxylate with eight propoxy groups, **c** isolated nonylphenol propoxylate with nine propoxy groups

**Fig. 2:**
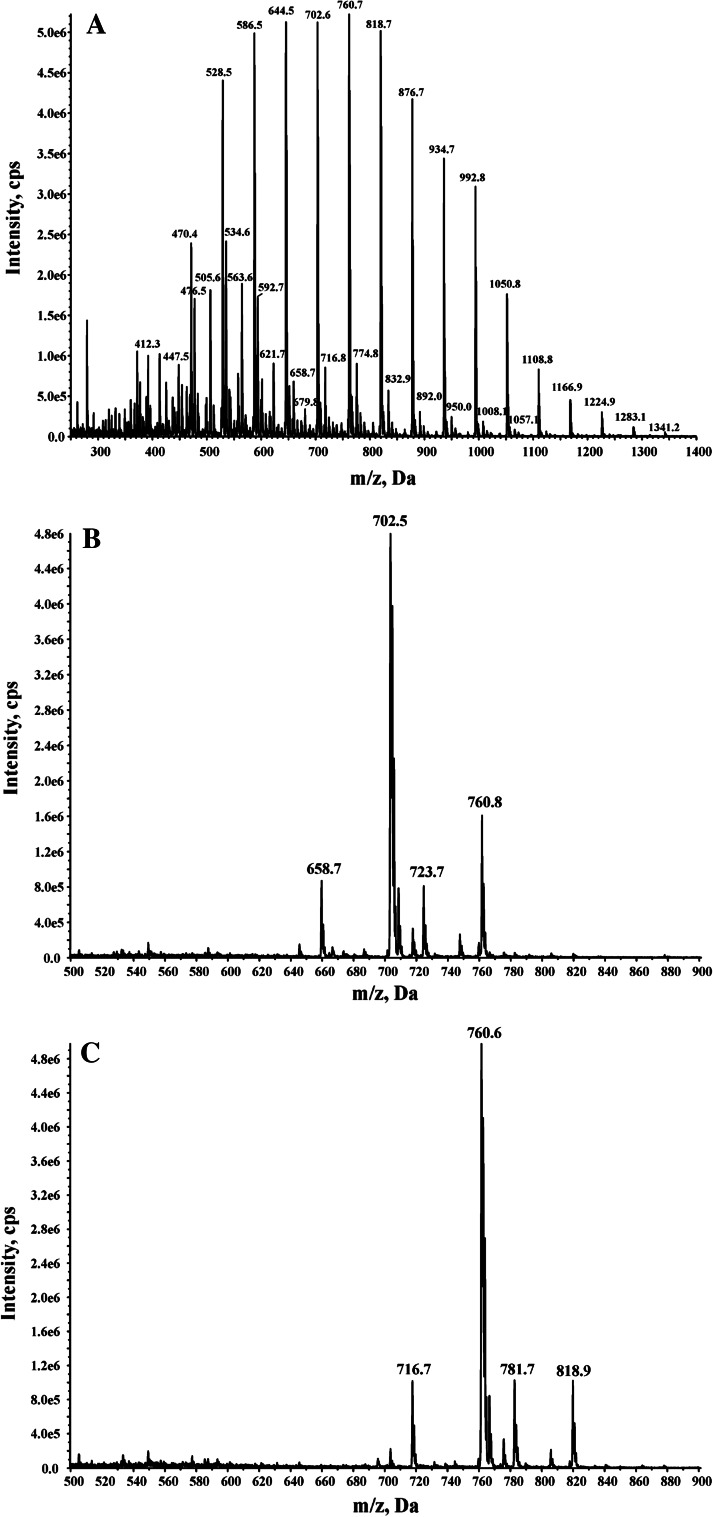
Mass spectra of nonylphenol propoxylates. Main peaks belong to ammonium adducts. **a** Spectrum of NPPOA10 used in the biodegradation test, **b** spectrum of isolated nonylphenol propoxylate with eight propoxy groups, **c** spectrum of isolated nonylphenol propoxylate with nine propoxy groups

Eluates from selected chromatographic peaks were collected for identification. Chromatograms of NPPOA10 and its selected homologues (NPPO8 and NPPO9) are presented in Fig. 1. Mass spectra of the entire analyzed mixture, as well as of the two selected homologues, are presented in Fig. 2. Composition of the analyzed mixture of NPPOs was calculated using a normalization procedure and assumption of equimolar signal response of particular homologues during their fluorescence detection.

Linearity of signal response was tested with the use of NPPOA10 at concentrations from 1 to 200 μg L^−1^. The highest injected concentration corresponded to twice the level of nonylphenol propoxylates used in the biodegradation test. The lowest concentration level corresponded to 1 % of the concentration used in the test, i.e. to 99 % of biodegradation. The correlation coefficients for all the homologues were higher than 0.995.

### HPLC–MS Analysis of Degradation Products

An UltiMate 3000 RSLC chromatographic system from Dionex was used for the analysis of degradation products of NPPOs. Each 5-μL sample was injected into a phenyl-hexyl column (50 mm × 3 mm I.D.; 1.8 μm) from Agilent Technologies (Santa Clara, CA, USA). The mobile phase employed in the analysis consisted of 5·10^−3^ mol L^−1^ ammonium formate in water and methanol at a flow rate of 0.5 mL min^−1^ at 35 °C. Gradient elution was performed by linearly increasing the percentage of organic modifier from 70 to 95 % in 15 min and then it was maintained at 95 % for 10 min. A pre-run time of 4 min was done before the next injection. The chromatographic system was connected to an API 4000 QTRAP triple quadrupole mass spectrometer from AB Sciex (Foster City, CA, USA). The LC column effluent was directed to the electrospray ionization source (Turbo Ion Spray). The Turbo Ion Spray source operated in positive ion mode and the mass spectrometer operated in scan mode. The following settings for the ion source and the mass spectrometer were used: curtain gas 10 psi, nebulizer gas 40 psi, auxiliary gas 40 psi, temperature 300 °C, ion spray voltage 4500 V, declustering potential 40 V.

### HPLC–MS Analysis of Nonylphenol

An UltiMate 3000 RSLC chromatographic system from Dionex was used. Samples of 5 μL were injected into a phenyl-hexyl column (50 mm × 3 mm I.D.; 1.8 μm) from Agilent Technologies. The mobile phase employed in the analysis consisted of 5·10^−3^ mol L^−1^ ammonium acetate in water and acetonitrile at a flow rate of 0.5 mL min^−1^ at 35 °C.

For the analysis of NP the following gradient was used: 0 min. 60 % acetonitrile; 3 min. 60 % acetonitrile; 5 min. 95 % acetonitrile; 8 min. 100 % acetonitrile. A pre-run time of 4 min was done before the next injection. The chromatographic system was connected to an API 4000 QTRAP triple quadrupole mass spectrometer from AB Sciex. The Turbo Ion Spray source operated in negative ion mode. The following settings for the ion source and the mass spectrometer were used: curtain gas 20 psi, nebulizer gas 40 psi, auxiliary gas 40 psi, temperature 480 °C, ion spray voltage −4,500 V, declustering potential −80 V and collision gas set to medium. The mass spectrometer operated in selected reaction monitoring mode. The dwell time for each mass transition was set to 100 ms. The quantitative transition was from 219.3 to 133.3 m/z at a collision energy set to −48 V and the confirmatory transition was from 219.3 to 147.3 m/z at a collision energy set to −35 V.

## Results and Discussion

### Selection of Solvents for Dispersive Liquid–Liquid Microextraction

Low water solubility of NPPOs made it necessary to use concentration techniques to perform a study on their biodegradation. Therefore, DLLME was used for the separation and concentration of NPPOs before HPLC analysis with fluorescence and mass spectrometric detection.

Selection of the extraction solvent and the dispersion solvent is a critical step in DLLME. There are several criteria to be met by the extraction solvent. Primarily, it has to give the possibility to extract the analytes. This solvent has also to be insoluble in water and heavier or lighter than water to enable phase separation after extraction of the analytes [16]. The main role of the dispersion solvent is to create dispersion of the extraction solvent in the water sample. Therefore, it has to be miscible with both the water sample and the extraction solvent. Use of several solvents has been reported in the literature including methanol, ethanol, acetone and acetonitrile [16]. Each of these solvents enables formation of proper dispersion. However, it should be taken into consideration, that each of these solvents can also influence the extraction process by changing the equilibrium in the system.

Five extraction solvents and four dispersion solvents were used in this study. As a result, twenty different combinations of solvents were tested, each in triplicate. Recovery and relative standard deviation (RSD) were calculated for 14 homologues of NPPOs, i.e. oligomers from NPPO3 to NPPO16. For optimization, the mean recovery for all tested NPPO homologues was calculated. The results obtained ranged from 18.0 to 61.7 %. Relative standard deviation values calculated for particular homologues were satisfactory, with 275 out of 280 values below 20 %. The relatively high mean recovery values (i.e. between 50.6 and 61.7 %) were found for tetrachloroethylene with all four dispersion solvents (with RSD for all homologues in these samples below 20 %). The highest among them was obtained for a mixture of tetrachloroethylene with ethanol. A similar result was obtained for a mixture of trichloroethane with ethanol (mean recovery 59.5 %). However, this combination of solvents had to be rejected because of problems with the availability of trichloroethane that emerged during the study. Therefore, tetrachloroethylene in ethanol was taken for further studies.

The chosen pair of solvents is different to that used for extraction of short-chained alkylphenol ethoxylates in a previous paper [12]. Thus, the exchange of ethoxy to propoxy groups in alkylphenol surfactants changes not only their properties and area of application but also influences the use of solvents taken for their extraction. Therefore, the choice of solvents used in DLLME is very important and must be made even for very similar analytes.

### Optimization of Dispersive Liquid–Liquid Microextraction Parameters

Optimization of solvent volumes and the effect of salt addition are further steps in the development of a final DLLME procedure. All these parameters can influence extraction of analytes from a sample solution. The volume of the dispersion solvent was tested from 0.5 to 4.0 mL. This range enables formation of a dispersion suitable for the microextraction process, as it was presented in the author’s previous studies on DLLME [12, 17, 18]. Mean recovery values were noted in the range from 46.4 to 83.5 %. RSD obtained for particular homologues was satisfactory. It exceeded 20 % for only one homologue in one test (NPPO16 for 0.5 mL of the dispersion solvent). The results obtained in this optimization show a gradual increase of mean recovery of NPPOs from 0.5 to 2.0 mL of ethanol. Then, a stable plateau can be observed from 2.0 to 3.0 mL with mean recovery in the range from 82.2 to 83.5 %. Further increases in the dispersion solvent volume lead to a decrease in recovery down to 78.4 %. As a result, 2.5 mL of ethanol was taken for further experiments.

The influence of the extraction solvent volume was tested in the range from 30 to 90 μL. Mean recovery was from 74.9 to 83.6 %. The RSD values were satisfactory. None of the homologues in these seven tests was analyzed with an RSD higher than 20 % and only two were analyzed with an RSD higher than 10 %. The highest mean recovery was noted in the range from 50 to 70 μL of the extracting solvent. Therefore, the volume of 60 μL was selected for further studies.

The influence of ionic strength was tested in the final step. Recovery of the analytes was examined with the addition of different amounts of sodium chloride. A range from 0 to 8 % of sodium chloride content was tested. The mean recovery of all NPPO homologues was about 80 % for all these samples and no increasing and decreasing tendency was found with the change of salt content. No improvement of extraction caused by the salting-out effect was observed. On the other hand, recovery of NPPOs was not lowered with the addition of salt in an amount much higher than the total dissolved solids level met in the biodegradation test. Thus, there was no salt added in further experiments.

Recovery obtained for particular homologues analyzed according to the final procedure ranged from 79.3 to 96.0 % and the relative standard deviation was from 2.0 to 7.6 %. These results together with high tolerance for salt presence enabled use of the developed method for the biodegradation studies of NPPOs.

### Biodegradation Test

**Fig. 3:**
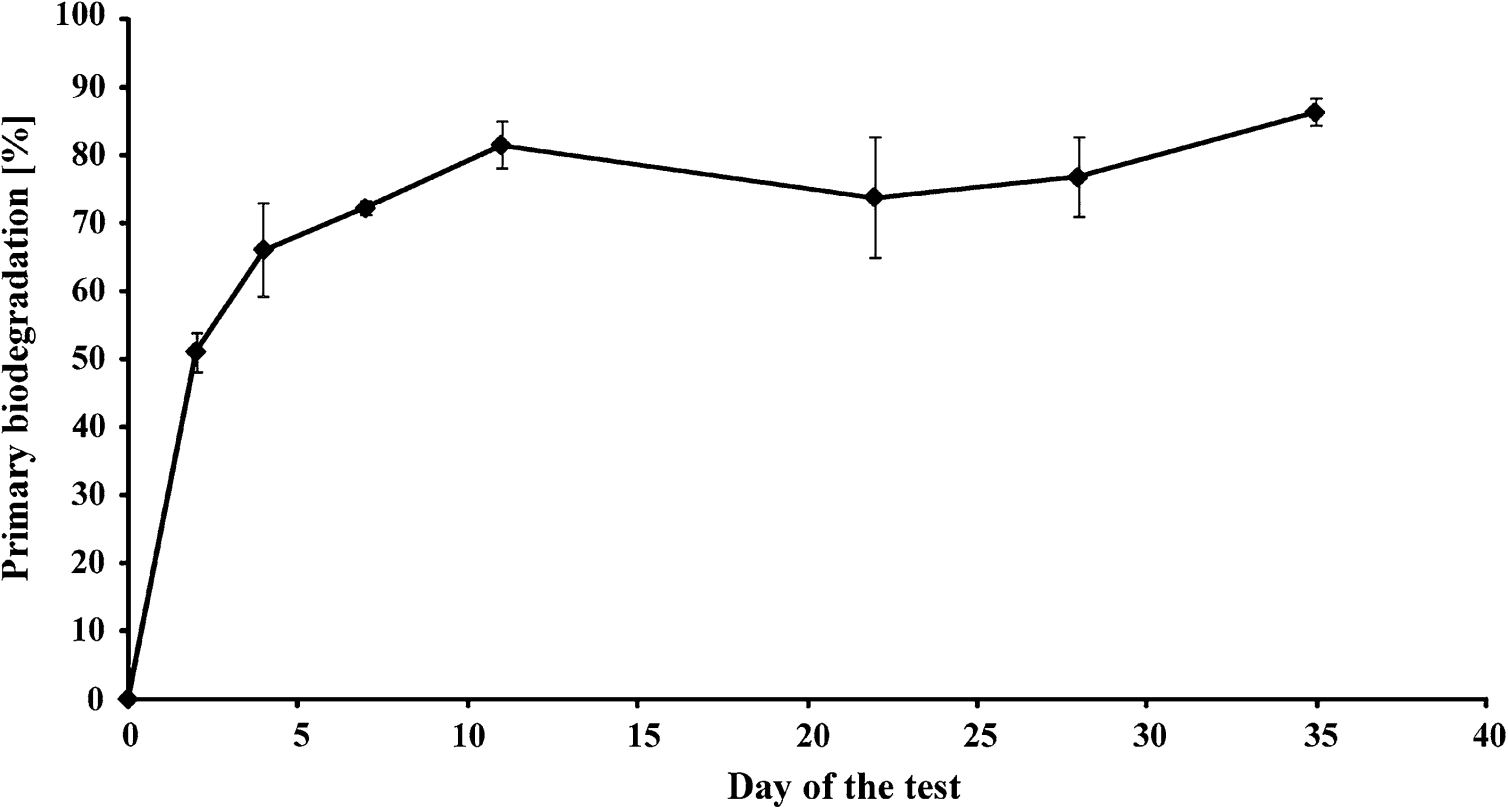
Primary biodegradation of nonylphenol propoxylates belonging to tested NPPOA10 surfactant (mean from three replicates with confidence intervals for a 95 % confidence level)

**Fig. 4:**
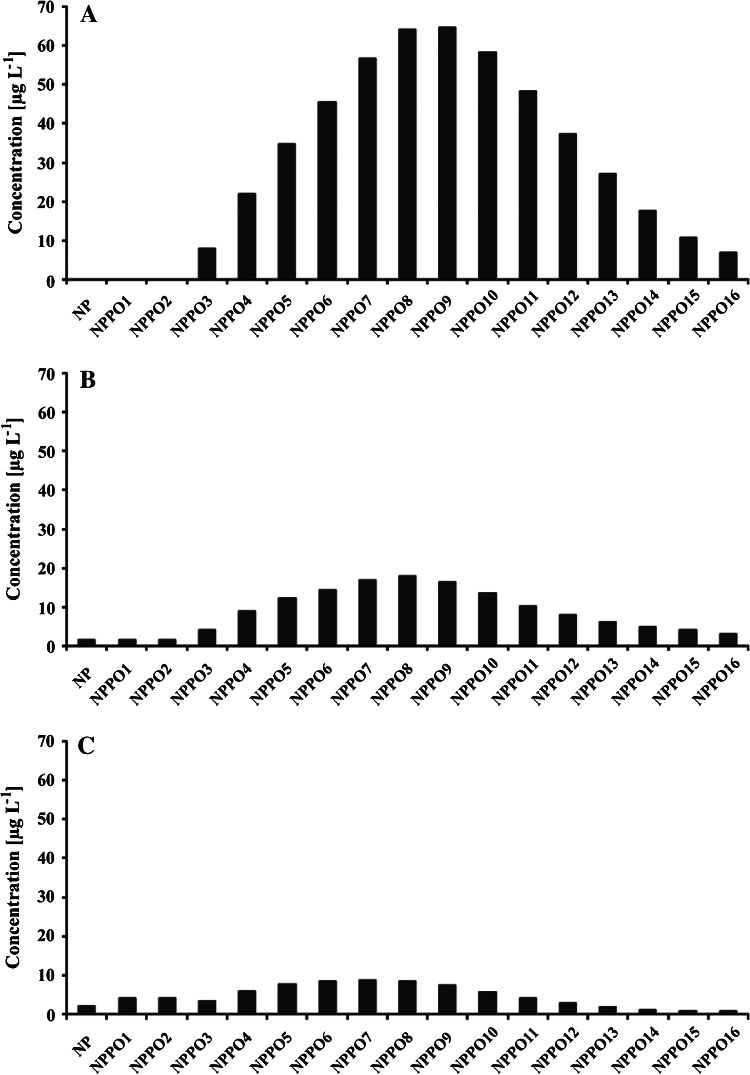
Concentration profile of homologues belonging to nonylphenol propoxylates during the biodegradation test. **a** Initial **b** on day 7 of the test **c** on day 35 of the test

Primary biodegradation of NPPOs is relatively fast. About 80 % of the homologues from the NPPOs subjected to the test had disappeared on the 10th day of the test (Fig. 3). However, it must be taken into consideration that this result cannot be directly compared with the limit given for ready biodegradable surfactants. The OECD requires 70 % of dissolved organic carbon removal during 28 days of the test. Primary biodegradation, measured in this study as disappearance of the NPPOs, leads to apparently faster biodegradation. Moreover, it must be also noted, that low water solubility of the NPPOs enforced use of a concentration lower than that proposed by the OECD [14]. This also can influence the biodegradation rate, i.e. accelerate the removal of NPPOs. Nevertheless, further disappearance of NPPOA10 seemed to stop as there was no considerable change observed on further days (Fig. 3). Also, the profile of homologues was not considerably changed during entire period of the study. There was always a maximum concentration noted for the same homologue in a series, i.e., for NPPO8 (Fig. 4). Lower homologues of NPPOs were formed during the test, but only in small amounts. This biodegradation scheme is similar to that observed during biodegradation of polypropylene glycols [6]. Also, the biodegradation schemes for NPPOs (noted here) and alkylphenol ethoxylates (by others) [19, 20] are similar to those of polypropylene glycols and polyethylene glycols [6], in that formation of lower homologues of propoxylates is preferred less to that of lower homologues of ethoxylates.

The missing biodegradation products of NPPOs could be observed in the chromatograms from the test as a group of coeluting peaks. Coelution with nonylphenol could not be excluded either. Therefore, screening for both the unknown compounds and the quantitative determination of nonylphenol were made with the use of high-performance liquid chromatography with mass spectrometric detection.

**Fig. 5:**
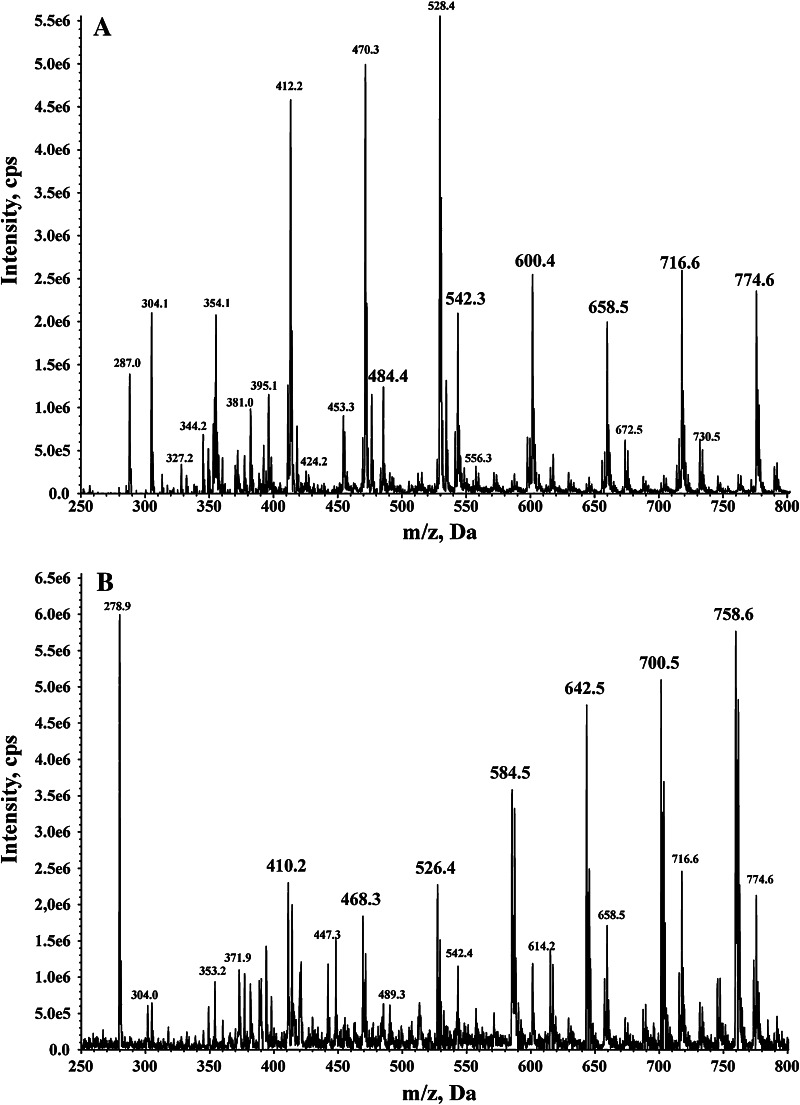
Mass spectra of oxidative biodegradation products of nonylphenol propoxylates extracted from several selected chromatographic peaks. **a** Series of ions characteristic to ammonium adducts of carboxylic acids marked with enlarged m/z values fonts **b** series of ions characteristic to ammonium adducts of ketones marked with enlarged m/z values fonts

Intensive peaks of two types of biodegradation products were observed in the chromatogram from the test samples. They can be attributed to both carboxylic acids and ketones (Fig. 5). Mass spectra extracted from the first group of selected chromatographic peaks reveal series of ions belonging to carboxylic acids at m/z = 484.4, 542.3, 600.4, 658.5, 716.6 and 774.6 (Fig. 5a). Mass spectra extracted from the second group of selected chromatographic peaks reveal series of ions belonging to ketones at m/z = 410.2, 468.3, 526.4, 584.5, 642.5, 700.5 and 758.6 (Fig. 5b). The carboxylic acids are analogues of carboxylated nonylphenol ethoxylates and are formed from NPPOs being primary alcohols. The ketones are formed during aerobic biodegradation of NPPOs being secondary alcohols. Both these groups of biodegradation compounds cannot be quantitatively analyzed, however, because their standards are not available.

**Fig. 6:**
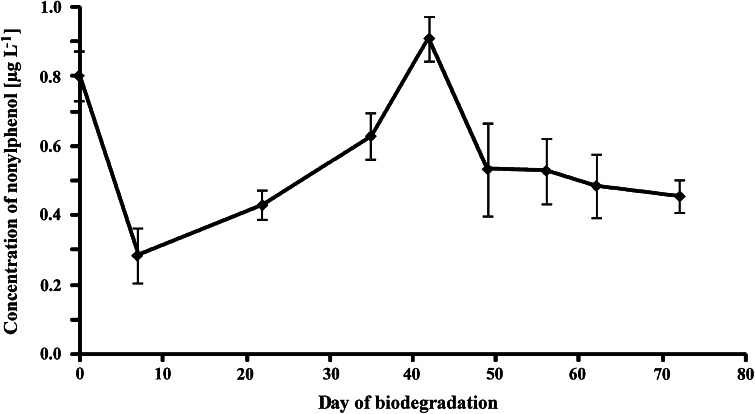
Concentration of nonylphenol during the biodegradation test of nonylphenol propoxylates (mean from three replicates with confidence intervals for a 95 % confidence level)

Presence of nonylphenol during the test was monitored with the use of mass spectrometric detection. It was present already at the beginning of the test at a concentration of 0.8 μg L^−1^ (Fig. 6), however, in a few days the biodegradation process led to a notable decrease in its concentration down to 0.3 μg L^−1^. Then, a gradual increase in nonylphenol concentration was observed up to 0.9 μg L^−1^ after over a month of biodegradation. Further biodegradation led to a decrease in nonylphenol concentration to a level of 0.4–0.5 μg L^−1^ as the biodegradation rate of nonylphenol was faster than its formation from NPPOs. The levels of nonylphenol analysed here were indeed very low and considerably lower than these noted in fluorescence detection of the test which confirms coelution with oxidative biodegradation products suggested above. Therefore, it can be stated that formation of nonylphenol from NPPOs is not favored during aerobic biodegradation. In contrast, the biodegradation of alkylphenol ethoxylates leads to the formation of considerable amounts of alkylphenol. Inclusion of a propoxy chain seems to block the biodegradation at a stage of formation of carboxylic acids and ketones. Therefore, a biodegradation scheme of NPPOs cannot be given yet and further studies have to be made to find biodegradation routes from carboxylic acids and ketones to the biomass.

On the other hand, a new alkylphenol-based surfactant has been introduced recently which should combine the positive properties of NPPOs and alkylphenol ethoxylates [21, 22]. This surfactant contains alkylphenol with one propoxy group and a number of ethoxy groups. On the basis of this and previous studies, it can be assumed that the ethoxy groups will enable rapid shortening of the polar chain and one propoxy group will block the formation of free alkylphenol. A new study will be commenced on this surfactant to confirm the above mentioned thesis.

## Conclusion

This paper presents a biodegradation study of NPPOs. Low water solubility of this surfactant made it necessary to develop a new isolation and concentration procedure. This was performed successfully with the employment of dispersive liquid–liquid microextraction. A suitable pair of extraction and dispersion solvents was selected and their amounts were optimized. The developed procedure was used to study the biodegradation of NPPOs.

Under the conditions used, NPPOs undergo primary biodegradation of over 80 % in 10 days. The biodegradation process leads to oxidation without non-oxidative shortening of the propoxy chain, which is in accordance with the biodegradation scheme observed for polypropylene glycols. Quantitative determination of these oxidative biodegradation products, is impossible however, due to the lack of appropriate standards. Also, their toxicological properties are unknown and should be a subject of further investigations.
